# Demineralization Pretreatments for Reducing Biomass Variability in Pyrolysis

**DOI:** 10.1021/acsomega.3c09321

**Published:** 2024-02-13

**Authors:** Carmen Branca, Colomba Di Blasi

**Affiliations:** † Istituto di Scienze e Tecnologie per l’Energia e la Mobilità Sostenibili (STEMS), C.N.R., P.le V. Tecchio, 80125 Napoli, Italy; ‡ Dipartimento di Ingegneria Chimica, dei Materiali e della Produzione Industriale, Università degli Studi di Napoli “Federico II″, P.le V. Tecchio, 80125 Napoli, Italy

## Abstract

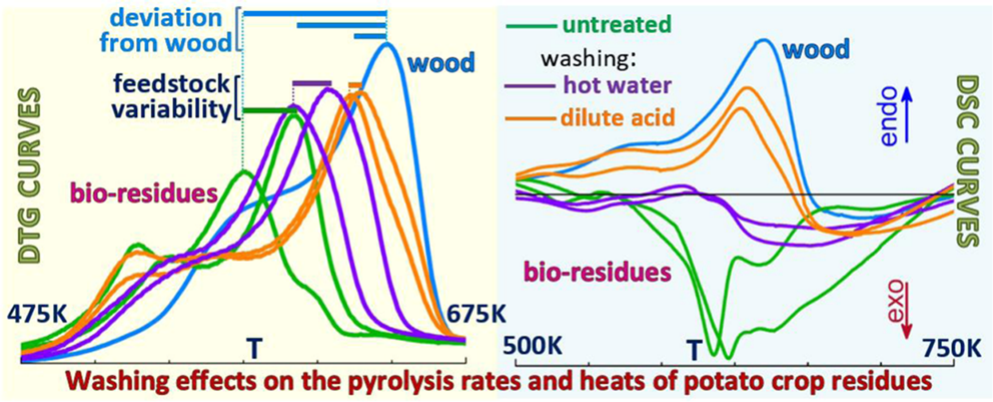

Thermogravimetric and calorimetric analyses are applied to study how washing modifies the pyrolysis rates and heats of five samples of potato plant stems. Hot (553 K) water or dilute (hydrochloric) acid washing of powdered samples causes a reduction in the alkali content by about 62–78 or 97–99%. The feedstock variability is highly reduced, especially for dilute acid treatment. The char yields drastically decrease up to 42–50%, with increases in the peak rates and corresponding temperatures of up to 20–60% and 50–60 K, respectively. Overall, these characteristic parameters closely approach the beech wood values used for comparison. The shape of the rate curves also testifies the dissolution of nonstructural organic components (pectin, starch, and protein) essentially to the advantage of holocellulose. The ratios between activation energy and order of the global devolatilization reaction increase from about 62–98 kJ/mol (no treatment) to 78–104 kJ/mol (hot water) and 113–124 kJ/mol (dilute acid) (versus 141 kJ/mol for wood). Following washing, the strong exothermic character of the crop residues (global reaction heats from −560 to −180 J/g) is lost. The pyrolysis becomes nearly thermally neutral after hot water washing (heats from −106 to −25 J/g). Furthermore, dilute acid washing makes the process shift from exothermic to endothermic with heats around 70–270 J/g (versus 238 J/g of wood).

## Introduction

The variability of biomass is a serious barrier for the scale-up and commercialization of thermochemical conversion technologies,^1^ in particular pyrolysis. A rough biomass classification is sometimes introduced^2^ as terrestrial or aquatic, but variations are huge even in the same class. For instance, the sector of origin of terrestrial biomass, such as energy crops or agricultural, forest, and industry waste, gives rise to largely different properties.^3^ The main structural components, hemicellulose, cellulose, and lignin, present contents and chemical characteristics varying from one biomass to another.^4−6^ The composition is further modified by nonstructural organic and inorganic phases. The organic part is often indicated under the item “extractives.” These consist of^4^ “various saccharides and carbohydrates, proteins, hydrocarbons, oils, aromatics, lipids, fats, starches, phenols, waxes, chlorophyll, resins, terpenes, terpenoids, acetyls, uronic acids, organic acids, sterols, glycosides, alkaloids, gums, mucilages, dyes, saponins, tannins, and flavonoids.” The content and nature are affected by the specific material and can reach rather high values, especially for agro-industrial residues, forestry wastes, and energy crops.^7,8^ High variability is also observed for the inorganic phase, depending on genetic and environmental issues and physiological and morphological differences among feedstock.^7^ Components primarily consist of^9^ potassium, calcium, sodium, magnesium, silicon, phosphorus, sulfur, chlorine, and generally, in minor amounts, others such as noble and heavy metals.

Although under practical conditions, physical processes control the pyrolytic conversion,^10−15^ the specific properties of each feedstock always play key roles. For instance, the packed-bed pyrolysis of a significant number of lignocellulosic biomasses^16,17^ clearly shows that, while the conversion times are essentially determined by the bulk density, the yields and quality of the products essentially depend on the chemical composition. This is understandable given the large diversity in the macrocomponent pyrolysis products^18^ and the catalysis of the inherent metals, in particular the alkali and alkaline earth metals (AAEMs), acting on the selectivity of primary and secondary reactions.^19−23^

Significant variations are also observed for the same type of biomass,^24−30^ depending on the genotype/cultivar, geographical origin, plant part, harvest year, and so on. Potato crop residue has been recently found^31−34^ to show very large variability in pyrolysis. Indeed, largely different composition, degradation characteristics, and exothermicity magnitude are observed at both the micro- and macroscale. For instance, for five samples originated from approximately the same geographical area, the factors of variations in the volatile products generated by the most important pseudocomponent (cellulose–starch), the global (exothermic) reaction heat, and, for packed beds, the maximum temperature overshoot are approximately in the range of 1.5–2.5^32,33^ (the corresponding range for the residue parts^34^ is about 1.3–1.4, excluding foliage). It is understandable that the large impact of variability on the pyrolysis characteristics makes difficult the optimization of the conversion systems for this kind of residue largely available worldwide.^35^ Hence, it is useful to look for possible feedstock pretreatments to make the chemicophysical properties more homogeneous and possibly more similar to those of wood. In fact, in addition to very high AAEM contents, for this waste, considerable amounts of pectin, starch, and protein are reported.^32^ Washing pretreatments, using water or dilute acid, have been demonstrated^36,37^ to remove, at different extent, the soluble parts of both organic and inorganic phases. However, their impacts on biomass variability have not yet been studied. The aspects related to changes in the reaction thermicity also deserve careful consideration as the endo- or exothermic character of lignocellulosic pyrolysis is an extremely important topic in pyrolysis but still largely unknown.^38,39^

In this study, the role of washing (hot water and dilute acid) is investigated on the pyrolysis and related energetic aspects for the potato crop residues, aimed at ascertaining whether feedstock variability can be reduced and the pyrolytic behavior can approach that typical of woody materials. The analysis is conducted at the microscale using thermogravimetric and calorimetric analyses. The main thermogravimetric parameters, the ratio between the activation energy and the order of the global devolatilization reaction, and the global pyrolysis heats are evaluated and compared with those of beech wood, which is used as a reference material.

## Materials and Methods

The materials investigated consist of potato plant (*Solanum tuberosum* L.) stems collected in the small geographical area of Irpinia (Sud Italy) at the end of their life cycle. Five samples (N.1–5) of different cultivars and harvest years are examined, with the same characteristics already presented in previous works^32,33^ (the sample N.5 is from a different batch, but the properties are roughly the same as the previous one). The variability, caused by cultivar and harvest year, was successfully depicted in terms of variable plant aging at the harvest time.^32−34^ So, to facilitate the comparison, the data of Table 1 and those presented and discussed in the following are listed from the least to the most aged item, that is, for the samples N.3, 4, 5, 1, and 2.

**Table 1:** (A, B) Proximate Analysis and AAEM Contents for the Untreated and hw- and aw-Washed Samples N.1–5^a^

(A) sample	VM [wt %]	FC [wt %]	ASH [wt %]
N.3	77.0	11.3	11.7
hw	86.3	10.1	3.6
aw	86.4	12.1	1.5
N.5	79.0	11.4	9.6
hw	83.0	14.0	3.0
aw	86.8	13.0	0.2
N.4	77.0	13.3	9.6
hw	85.0	10.0	5.0
aw	87.0	11.3	1.7
N.1	77.1	11.2	11.7
hw	86.3	10.8	2.9
aw	88.0	11.0	1.0
N.2	76.9	12.6	10.5
hw	86.4	9.3	4.3
aw	86.5	11.9	1.6
beech wood	86.5	13.1	0.4

(B) sample	K [ppm]	Ca [ppm]	Mg [ppm]	Na [ppm]
N.3	75 030	12 130	1613	175
hw	12 717	12 120	181	73
aw	591	2460	47	12
N.5	73 800	5768	1860	236
hw	1354	5541	1091	157
aw	191	70	53	67
N.4	50 320	12 200	324	242
hw	10 939	12 577	278	98
aw	109	880	12	53
N.1	82 930	10 770	3864	323
hw	16 134	8685	2208	98
aw	383	1210	8	25
N.2	58 420	15 590	3999	339
hw	5617	12 573	1143	113
aw	121	1288	73	59
beech wood	796.4	584	221.6	76.8

a Beech wood data is included for comparison.

As in previous studies,^25,28,40−45^ washing of powdered material (sizes in the range of 50–100 μm) is carried out using either hot water (hw) at a temperature of 353 K or dilute acid (also indicated in the following as acidic water, aw) of 0.1 mol/L HC1 at ambient conditions. The feedstock to solvent ratio is taken equal to 0.01 g/mL (initial sample mass around 0.5–1 g), with treatment times of 2 h (hw) or 4 h (aw). The suspensions are filtered by means of a 40 μm metallic wire net, and for aw washing, the collected material is washed with distilled water until neutrality, followed by drying at 353 K.

The untreated and washed residues are characterized in terms of proximate analysis^28,32−34^ and inductively coupled plasma mass spectrometry (ICP-MS) (Agilent 7500ce) analysis of the alkali metals. The decomposition characteristics are evaluated using integral (TG, mass fraction, *Y*) and differential (DTG, time derivative of the mass fraction, −d*Y/*d*t*) thermogravimetric analyses (Mettler TGA 1) with a pulverized (sizes below 100 μm) sample mass of 5 mg, heated at 5 K/min up to 773 K, under a nitrogen flow of 50 mL/min, including a predrying stage at 383 K for 30 min. The characteristic temperatures, rates, and mass fractions^25,28,46^ are evaluated and compared for the various cases.

Accurate kinetic characterization requires the introduction of multistep reaction schemes, to describe macrocomponent dynamics, and parameter fitting methods, as done^32^ for the untreated feedstocks under exam. However, for engineering evaluations, analytical approaches are available, assuming a single n-order reaction based on mathematical relationships between thermogravimetric and kinetic parameters. More specifically, the mathematical treatment proposed in refs (47,48) to evaluate the ratio between activation energy and reaction order, *E*/*n*, is used here for both untreated and washed feedstocks. To apply this analysis, the integral and differential weight loss curves are expressed in terms of conversion, defined as α = (1 – *Y*)/(1 – *Y*_773_), with the corresponding dα*/*d*T*, where *Y*_773_ is the char yield at complete conversion, assumed to coincide with the mass fraction measured at a temperature of 773 K.^25,28^ The mathematical expression of the *E*/*n* ratio is the same as in refs (47,48) (see eq 4 of ref (48)) and uses the thermogravimetric parameters related to the peak rate.

Differential scanning calorimetry (DSC) curves (calorimeter Mettler DSC 1/700) are measured together with the corresponding weight loss data, again using pulverized samples as above, for the untreated and washed feedstocks. A sample mass of 5 mg is heated at 20 K/min up to 773 K, under a nitrogen flow of 160 mL/min, including a predrying stage at 383 K for 20 min, in aluminum open crucibles. The heat reaction curves are computed following the same method previously used,^49−54^ including a radiation correction for open crucibles. To proceed with the computation, the equations are required for the specific heats of wood^49^ and corresponding char^55^ and of crop residues and corresponding chars.^52^ Then, the global reaction heat, *H*, is computed by means of numerical integration of the heat flow curves. The thermogravimetric and calorimetric curves are measured in triplicate, showing good repeatability, as already observed for the untreated samples^32^ and other feedstocks.^28,56,57^

Beech wood, a sort of standard woody biomass, is used for comparison of both the thermogravimetric and calorimetric behavior of the untreated and washed food crop residues. Wood pretreatments are not made. In fact, as already stated above, the scope of the investigation is to ascertain whether washing acts to reduce the variability of the residues and to bring their behavior near that of wood.

## Results and Discussion

The effects of the washing pretreatments are first examined following the application of both hot and acidic water. Then, the changes caused on the thermogravimetric parameters, the ratio *E*/*n* (activation energy/reaction order) of the global devolatilization reaction, and the global reaction heats are examined.

### Proximate Analysis and Alkali Content

The volatile matter (VM), fixed carbon (FC), and inorganic (ASH) contents and the alkali metal (K, Ca, Mg, and Na) contents are summarized in Table 1A,B (data for beech wood is included for comparison^33,44^). The proximate analysis results show values around 77–79 (VM), 11–13 (FC), and 10–12 (ASH) wt % for the untreated samples N.1–5. The corresponding contents of alkali metals are in the range of 6.3–9.8 wt %. Both washing pretreatments act to reduce or eliminate the soluble part of the inorganic content^36,37^ as well as the possible soil contamination of the samples. Consequently, in the first place, the alkali catalysis on the decomposition reactions, generally favoring charring with respect to devolatilization, is reduced or eliminated, and second, the amount of active material is increased. Together with demineralization, nonstructural soluble organic compounds are also removed to a certain extent.^36,37^ Hence, the active material, constituting the samples, changes in relation to both contents and chemical properties.

The results listed in Table 1B show that the content of alkali metals is highly reduced or practically eliminated after the pretreatments (reductions in the total AAEMs around 62–78 and 97–99 wt % for the hw or the aw washing, respectively). Reductions in the total ash (Table 1A) range from 48 to 75 or 82 to 98 wt % again for the two treatments in the same order. For the hw treatment, the reduction factors, for the most abundant metal, K, vary between about 5 and 10 (factors below 3.5 for the much less abundant Mg and Na), whereas the Ca content is left practically unvaried. It is plausible that, in some cases (samples N.2 and N.4), the ash consists of a considerable part of components that are not soluble in water; for instance, silicon that anyway does not affect the pyrolysis reactions.^37^

Following washing, the VM content always increases with values around 83–86 or 86–88 wt % for the hw and the aw washing, respectively. To understand the trend shown by the FC contents, it should be kept in mind that the conditions of proximate analysis^28,32,33^ do not require a kinetic control and that alkali metals play a complex role in conversion. In fact, they catalyze charring reactions so that char formation is favored. However, they also enhance the pyrolysis exothermicity,^20,21^ leading to higher actual reaction temperatures, which are detrimental to char formation. Therefore, the practically complete alkali removal for the aw treatment, by lowering the amount of heat released, generally results in higher FC values. For the hw treatment, the FC contents generally are lower, owing to still non-negligible alkali contents, associated with approximately invariant temperature enhancement. In fact, the exothermicity magnitude during pyrolysis of potassium-loaded wood remains at its maximum for additive contents above 2 wt %.^20,21^ Washing makes closer to the proximate analysis and alkali contents of the agricultural residues and wood, especially for the aw treatment.

### TG-DTG Curves of the Demineralized Samples

**Figure 1:**
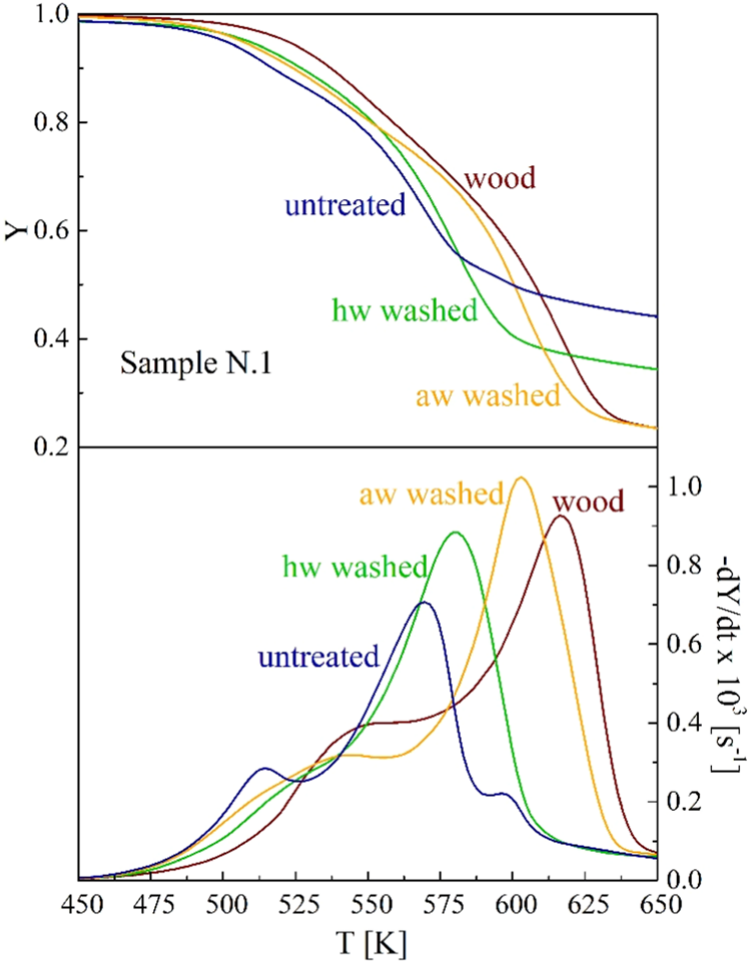
Thermogravimetric curves of the untreated, the hw-washed, and the aw-washed samples N.1 versus the heating temperature (heating rate 5 K/min). Beech wood curves are included for comparison.

**Figure 2:**
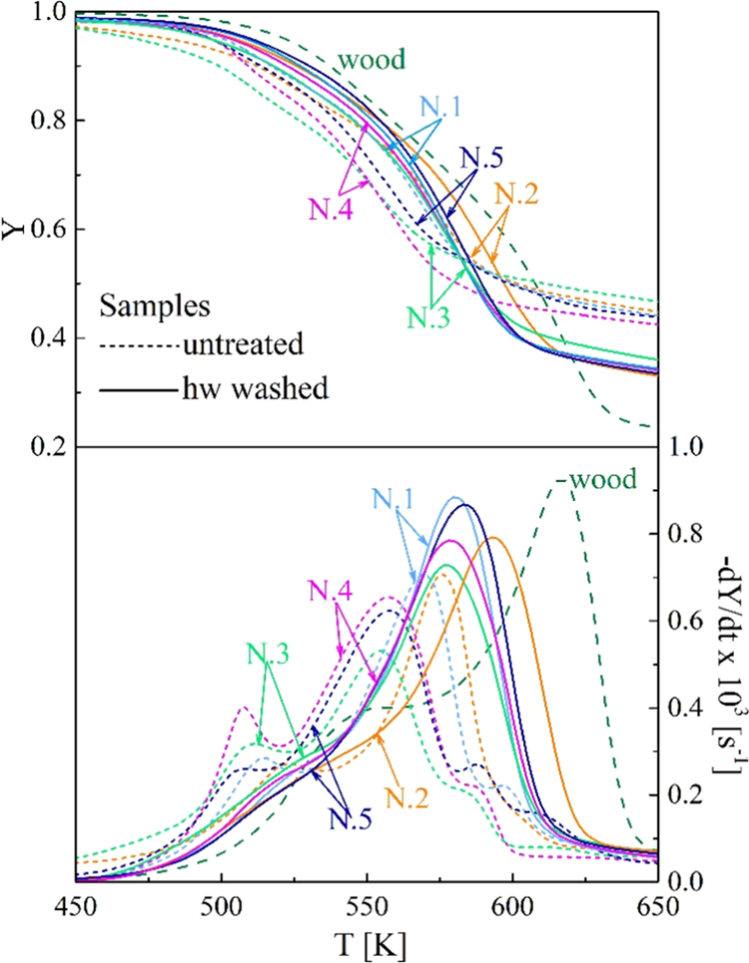
Thermogravimetric curves of the untreated and the hw-washed samples N.1–5 versus the heating temperature (heating rate 5 K/min). Beech wood curves are included for comparison.

**Figure 3:**
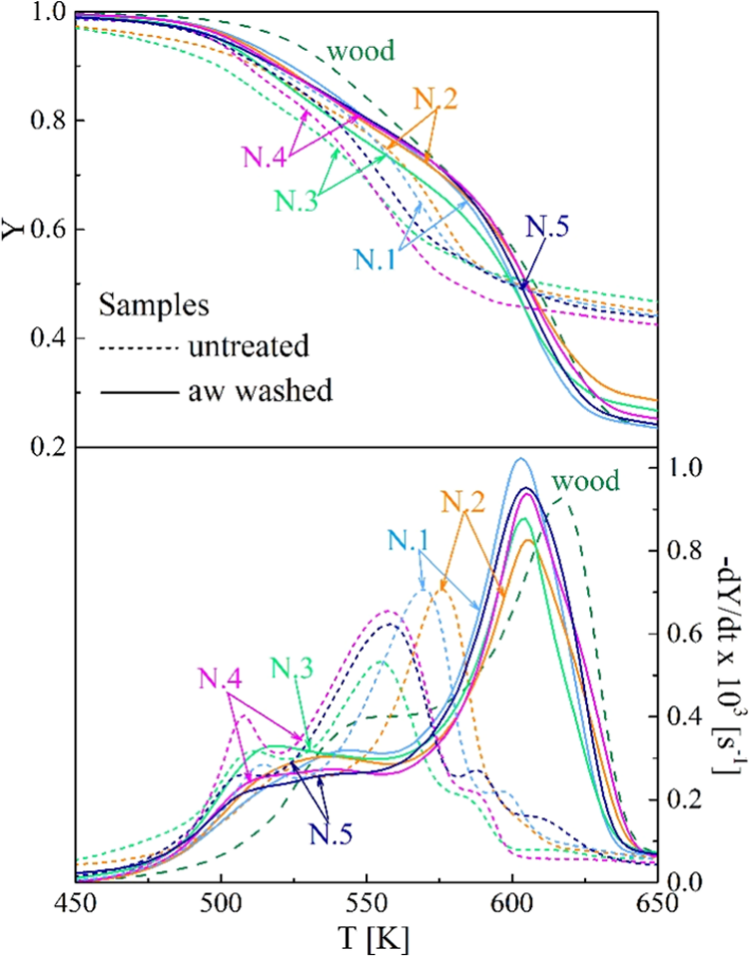
Thermogravimetric curves of the untreated and the aw-washed samples N.1–5 versus the heating temperature (heating rate 5 K/min). Beech wood curves are included for comparison.

The integral (TG) and differential (DTG) thermogravimetric curves are compared in Figure 1 for the untreated and the hw- and aw-washed samples N.1, where the beech wood curves are also reported. Figures 2–3 provide a general overview of the effects of the hw and aw treatments on the thermogravimetric behavior of samples N.1–5. The TG-DTG curves of lignocellulosic biomass decomposition, in particular woody materials, are characterized by three main zones: a shoulder, a peak rate, and a tail, mainly associated with hemicellulose, cellulose, and lignin dynamics. In general, there is an overlap among the macrocomponent rates so that the introduction of pseudocomponents is required for kinetic modeling.^11,12^ The weight loss curves of potato crop residues show more complex dynamics, owing to the presence of large quantities of extractives, pectin, starch, and protein, and the significant AAEM amounts that enhance the overlap.^32^ The different zones of the weight loss curves can be associated with the dominant component, considering the temperature range where the corresponding model compounds decompose. Excluding the pseudocomponent “light extractives,” owing to the low amounts of volatiles they release, in the absence of pretreatment, three main pseudocomponents or zones of the weight loss curves are introduced^32^ and used in the following. They are pectin–hemicellulose (first or low-temperature zone), cellulose–starch (second or intermediate-temperature zone), and lignin–protein (third or high-temperature zone).

Washing effects are qualitatively the same for all the samples N.1–5 and similar to those observed for other materials.^25,28,40−45,56^ The most evident change consists of an increase in the peak rates and the corresponding temperatures with an improved separation between the reaction zones. The aw washing effects are quantitatively higher. Furthermore, there are important changes in the shape of the rate curves. To partly justify the new trends, it is useful to remind that the leachates from washing treatments decompose over the same temperature range as the origin material, as observed for several biomass materials.^25,58−60^ The low-temperature rates become slower, and the peak generally disappears. This is due to the loss of pectin and other thermally labile components. As already observed, for the second zone, the higher absolute peak rate moves at higher temperatures, testifying to an increase in the cellulose content and its crystallinity degree. The increased crystallinity can be attributed to the reduction or removal of alkalis^61^ and starch, which is an amorphous substance. The high-temperature peak rate (third zone) is not clearly visible any longer, not only for the partial dissolution of phenolic compounds and proteins. In fact, for the hw washing, it is most likely hidden by the evolution of the second-zone components occurring at higher temperatures. For the aw washing, it is barely visible under the swollen part of the decay zone after the absolute peak rate. From the physical point of view, this feature can be explained considering that the catalysis of alkali metals on lignin decomposition is smaller than that exerted on the cellulose component.^23^ Thus, only if they are almost completely removed, such as for the aw treatment, the lignin decomposition significantly moves toward higher temperatures. The peculiar shape of the rate curve might also be due to the specific properties of the lignin whose chemical (and degradation) characteristics are remarkably dependent upon the origin materials (e.g., woods and straws^62,63^). Overall, following the washing pretreatments, the rate curves tend to qualitatively approach those of woody biomass (a shoulder, a peak, and a tailing zone).

The influences of the pretreatment on the thermogravimetric characteristics can be quantified from the parameters^25,28,46^ listed in Table 2 (definitions in Figures SM1 and SM2 of the Supplementary Material (SM)). They include the peak rate, −d*Y*_peak_/d*t*, and the peak/shoulder rate of the first or third zone, −d*Y*_ps1_/d*t* or d*Y*_ps2_/d*t*, with the corresponding temperatures *T*_peak_ and *T*_ps1_ or *T*_ps2_ and mass fractions, *Y*_peak_, and *Y*_ps1_ or *Y*_ps2_. It should be specified that the −d*Y*_ps2_/d*t* rate can be defined only for the untreated and the aw-treated samples, where a third reaction zone (or at least its final part) can be identified. In the former case, a local peak rate is observed, whereas in the latter, the characteristic point corresponds to the first minimum or the shoulder of the d^2^*Y*/d*t*^2^ (the shoulder is defined by a nearly zero value of the third time derivative of the mass fraction). For this treatment, there is also an additional characteristic point, at higher temperatures, again identified by the same features as above, corresponding to the conclusion of the third reaction zone (this is reported in parentheses in Table 2).

**Table 2:** Thermogravimetric Parameters (Heating Rate 5 K/min) for the Untreated and hw and aw-Washed Samples N.1–5^a^^,^^b^

sample	–d*Y*_peak_/d*t* × 10^3^ [s^–1^]	–d*Y*_ps1_/d*t* × 10^3^ [s^–1^]	–d*Y*_ps2_/d*t* × 10^3^ [s^–1^]	*Y*_peak_	*Y*_ps1_	*Y*_ps2_	*Y*_773_
N.3	0.54	0.32	0.21	0.66	0.86	0.55	0.39
hw	0.73	0.30		0.59	0.85		0.28
aw	0.88	0.33	0.70	0.46	0.91	0.44	0.20
N.5	0.62	0.26	0.27	0.68	0.91	0.53	0.39
hw	0.87	0.22		0.56	0.92		0.27
aw	0.95	0.26	0.74	0.48	0.83	0.34	0.18
N.4	0.65	0.38	0.22	0.63	0.90	0.48	0.36
hw	0.78	0.26		0.58	0.89		0.27
aw	0.94	0.27	0.80	0.51	0.84	0.42	0.19
N.1	0.71	0.28	0.22	0.64	0.91	0.51	0.38
hw	0.88	0.29		0.56	0.88		0.27
aw	1.00	0.32	0.85	0.47	0.83	0.37	0.17
N.2	0.71	0.27	0.15	0.63	0.87	0.50	0.38
hw	0.79	0.26		0.53	0.88		0.25
aw	0.82	0.30	0.64	0.49	0.85	0.40	0.22
beech wood	0.92	0.42		0.41	0.80		0.18

sample	*T*_peak_ [K]	*T*_ps1_ [K]	*T*_ps2_ [K]	fwhm [K]	Δ*T*_1_ [K]	Δ*T*_2_ [K]
N.3	555	511	582	70	50	29
hw	577	532		51	50	34
aw	604	519	611 (623)	40	34	31
N.5	558	506	588	48	54	27
hw	584	523		50	53	23
aw	605	534	617 (625)	43	35	30
N.4	557	508	586	53	52	26
hw	579	525		51	50	31
aw	604	538	613 (631)	42	31	39
N.1	570	513	597	41	46	19
hw	580	533		42	45	27
aw	603	544	612 (622)	40	34	31
N.2	576	523	603	34	33	19
hw	593	534		51	48	30
aw	606	536	617 (629)	44	38	34
beech wood	616	558		48	47	22

a Peak rate, −d*Y*_peak_/d*t*, and peak/shoulder rate of the low- and high-temperature zones, −d*Y*_ps1_/d*t* and −d*Y*_ps2_/d*t*, with the corresponding temperatures, *T*_peak_ and *T*_ps1_, and *T*_ps2_, and mass fractions, *Y*_peak_, and *Y*_sp1_ and *Y*_sp2_, temperature range *fwhm*, the final charred residue, *Y*_773_, and the temperature intervals Δ*T*_1_ and Δ*T*_2_ (in bracket, for the variable *T*_ps2_, the second characteristic temperature of this zone is reported).

b Beech wood data is included for comparison.

The temperature range fwhm (the full width of the rate curve at the half-maximum) and the final charred residue (mass fraction) at a temperature of 773 K, *Y*_773_, are also considered. Finally, as already done for the analysis of lignocellulosic char oxidation curves,^64^ the characteristic temperature ranges determined by the position of the peak rate and the intercepts (on the temperature axis) obtained by extrapolating the tangents at the points of the fwhm, Δ*T*_1_ and Δ*T*_2_, are also evaluated. This data gives information about the skewness of the rate curves, which influences the kinetic parameters of the global devolatilization reaction.

**Figure 4:**
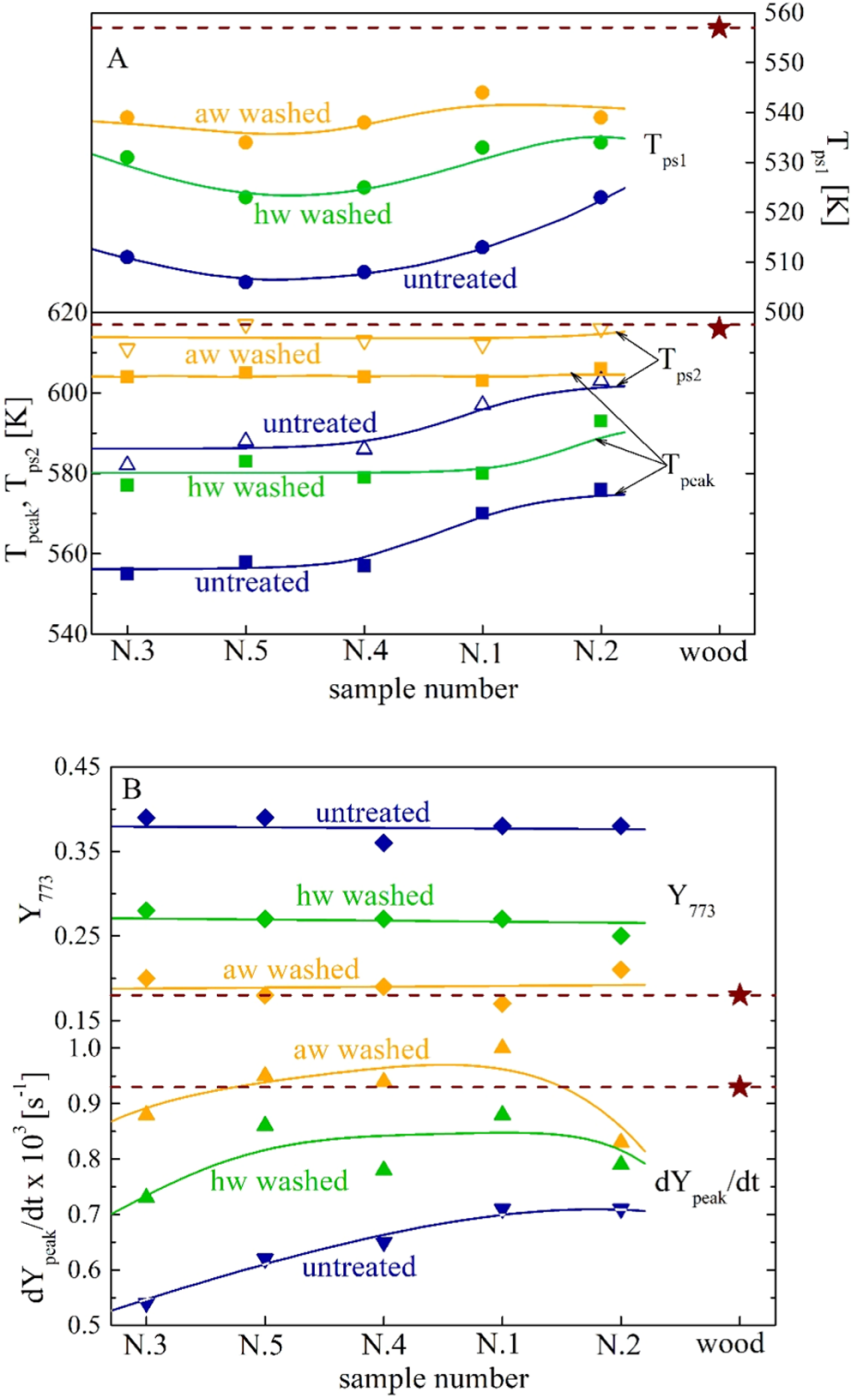
(A, B) Effects of the hw and the aw washing on the thermogravimetric parameters *T*_ps1_, *T*_peak_, *T*_ps2_ (A) and *–*d*Y*_peak_/d*t*, *Y*_773_ (B) for the samples N.1–5 (the data is plotted from the least to the most aged sample). Beech wood data is included for comparison.

**Figure 5:**
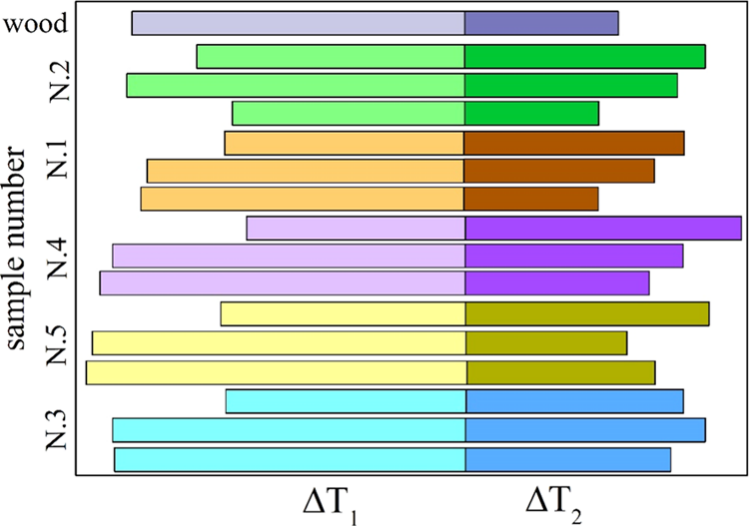
Histograms of the temperature differences Δ*T*_1_ and Δ*T*_2_ for the untreated, the hw-washed, and the aw-washed samples N.1–5 (the data is plotted from the least to the most aged sample). Beech wood data is included for comparison.

**Figure 6:**
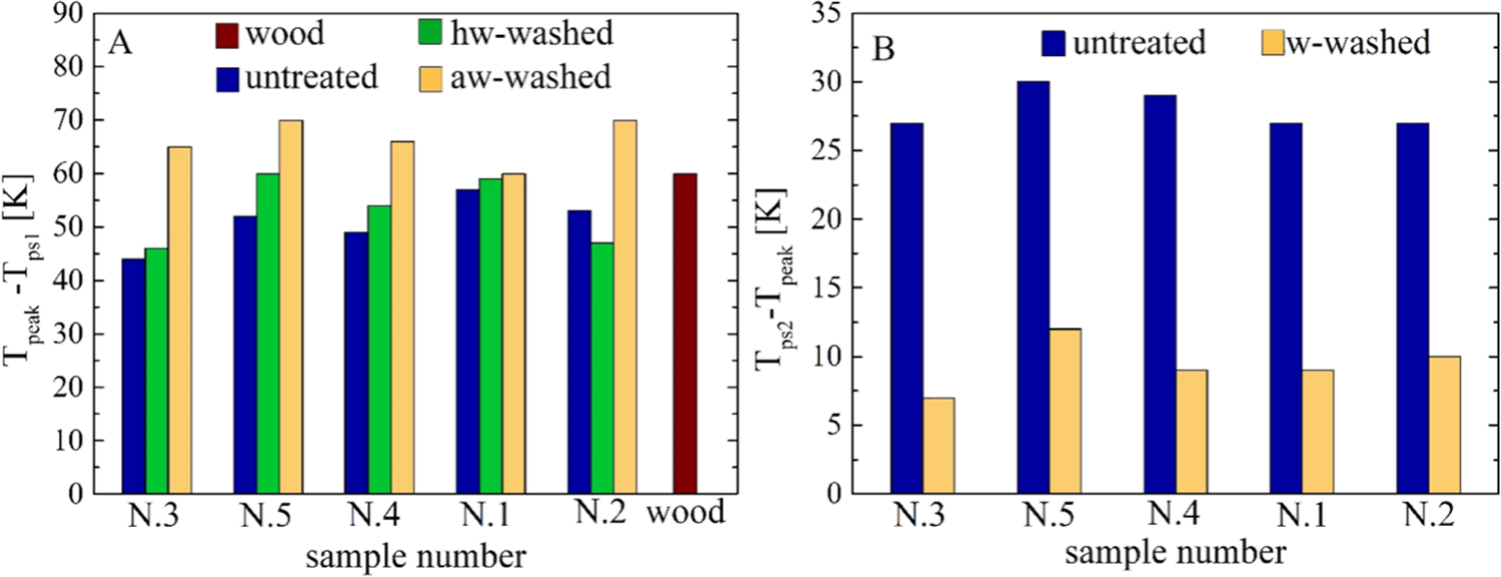
(A, B) Histograms of the temperature differences (*T*_peak_ – *T*_ps1_) (A) and (*T*_ps2_ – *T*_peak_) (B) for the untreated, the hw-washed, and the aw-washed samples N.1–5 (the data is plotted from the least to the most aged sample). Beech wood data is included for comparison.

The main parameters are also shown in Figure 4A,B (*T*_ps1_, *T*_ps2_, *T*_peak_, *–*d*Y*_peak_/d*t*, and *Y*_773_) and the histograms in Figure 5 (Δ*T*_1_ and Δ*T*_2_). Some characteristic differences (*T*_peak_ – *T*_ps1_, *T*_ps2_ – *T*_peak_) are also represented by the histograms in Figure 6A,B.

As already noticed, quantitative differences are significant between the effects of the two pretreatments, with the acidic treatment being the most important. Also, the pretreatments exert a stronger effect on the less aged samples N.3, 4, and 5, owing to the larger amounts of soluble organic components.^32^ Following washing, the peak rates increase significantly (up to factors of 20–60%). For this parameter, the differences among the five samples are reduced to about 20% with respect to 30% in the absence of treatment. The mass fraction, in correspondence with the peak rate, becomes smaller as the washed active material preferentially leads to volatile product formation. Indeed, the yields of char decrease to about 42–55%. The temperature *T*_peak_ also increases, from values in the range of 555–576 K (no pretreatment) to 577–593 and 603–606 K, for the hw or aw treatments. Again, washing reduces the differences among samples, with variations for this parameter decreasing from 21 K (absence of pretreatments) to 16 and 3 K for the treatments in the same order as above.

For the first reaction zone, the temperatures of the peak or shoulder rates, *T*_ps1_, again increase (from 512–529 K (no treatment) to 525–533 and 545–550 K for the hw and aw washing in the order), progressively reducing the differences among the samples (from 17 to 8 and 5 K). However, the displacement toward higher values of this first zone is less important than that of the second zone discussed above. Indeed, the differences *T*_peak_ – *T*_ps1_ vary from about 39–51 K (absence of treatment) to 44–60 and 53–59 K for the hot and acidic water washing. That is, the alkali effects on cellulose decomposition are stronger than those on the hemicellulose. Hence, washing causes a reduction in the overlap between the two adjacent reaction zones. The analysis of the third reaction zone is more complicated because, as was already observed, washing causes the disappearance of the local peak rate. Moreover, a change in the slope of the rate curve can be identified only for the aw washing. As expected, *T*_ps2_ becomes higher and the differences among samples are reduced (values of 611–617 K versus 555–576 K of the untreated sample). Moreover, the differences *T*_ps2_ – *T*_peak_ are reduced (7–12 vs 26–30 K), confirming that the effects of alkali removal are more important for cellulose than lignin. Finally, the second characteristic temperature of the third zone varies between 623 and 631 K.

Washing affects the fwhm parameter through changes in the composition of the active material and the influence of alkali metals on the position of the peak rates. As for the latter, the mutual overlap between the decomposition zones of hemicellulose and cellulose is always reduced. That between the cellulose and lignin zones slightly decreases or increases for the hw or the aw washing, respectively. On the other hand, the removal of significant amounts of organic matter, modifying the shape of the rate curves, also modifies the conditions leading to the definition of fwhm. Overall, a tendency of wider fwhm is observed. The untreated sample N.3 owns the widest value in the absence of treatment due to the large contents of pectin making possible the attainment of high rates already at low temperatures (70 K versus 40–50 K for the treated samples). However, it is useful to observe that, following washing, the differences among the samples are again reduced with fwhm variations among samples from 36 K to 15 or 6 K. Despite the swelling of the right side, the skewness of the rate curves is generally from the left side, as indicated by Δ*T*_1_ > Δ*T*_2_. Washing generally makes Δ*T*_2_ increase (the sample N.2 deviates from this trend). Δ*T*_1_ remains roughly the same or also increases (hw or aw washing; deviations again for the sample N.2).

**Figure 7:**
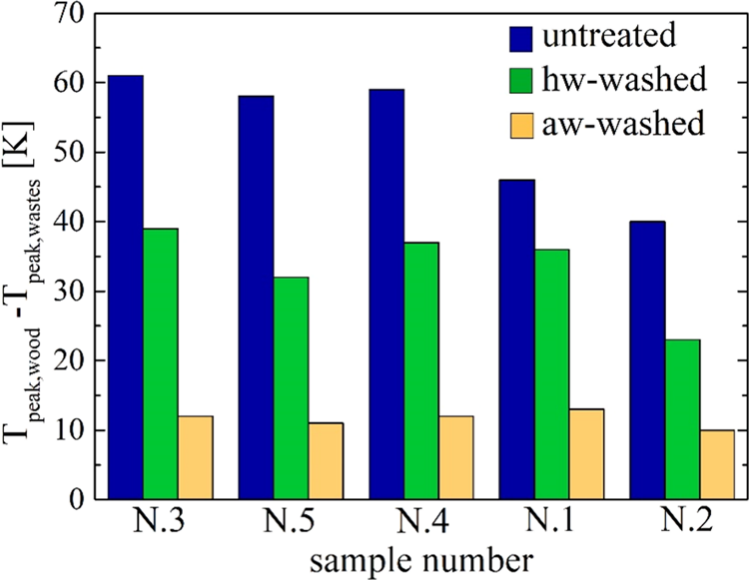
Histograms of the differences between the *T*_peak_ temperature of beech wood and those of the untreated, the hw-washed, and the aw-washed samples N.1–5 (the data is plotted from the least to the most aged sample).

As already noticed for the proximate analysis, washing makes the residues more similar to wood, especially for the aw washing. For the untreated agricultural residues, the most aged ones (N.1–2) are those more like beech wood, though quantitative differences are anyway large (overall, *T*_peak_ lower by about 40–60 K). As shown by the histograms in Figure 7, following hot water washing, sample N.2 is still the most similar to wood, while the others also move closer (*T*_peak_ differences of 24 K for sample N.2 and 33–40 K for the others). However, the rate curves are more swollen for the decay part. This feature is even more evident for the aw-treated samples, whose peak rate position approaches closer to that of beech wood (*T*_peak_ differences around 5–10 K). Moreover, the peak rates and the char yields also become comparable. These findings support the speculation that for the aw-treated residues the cellulose contents and properties become approximately the same as for wood and that differences may be attributed to the different nature of lignin and most likely to differences in the hemicellulose as well.

In summary, the thermogravimetric data shows that the feedstock variability is reduced by the washing pretreatment, with changes that are stronger for the less aged samples N.3–5. The behavior of these approaches the same as that of the most aged ones N.1–2. Overall, the washed residues tend to exhibit thermogravimetric curves that become both qualitatively and quantitatively close to that of (untreated) beech wood, a consequence of more similar chemical compositions. The ash removal causes the reaction process to proceed at higher temperatures. The changes in the shape of the washed residues, becoming more like wood, indicate that a large part or the entire amount of nonstructural organic matter is also eliminated. The increase in the peak rate and strong reduction in the char yields support the speculation that the holocellulose content increases.

### Analytical Evaluation of the Thermogravimetric Curves

**Figure 8:**
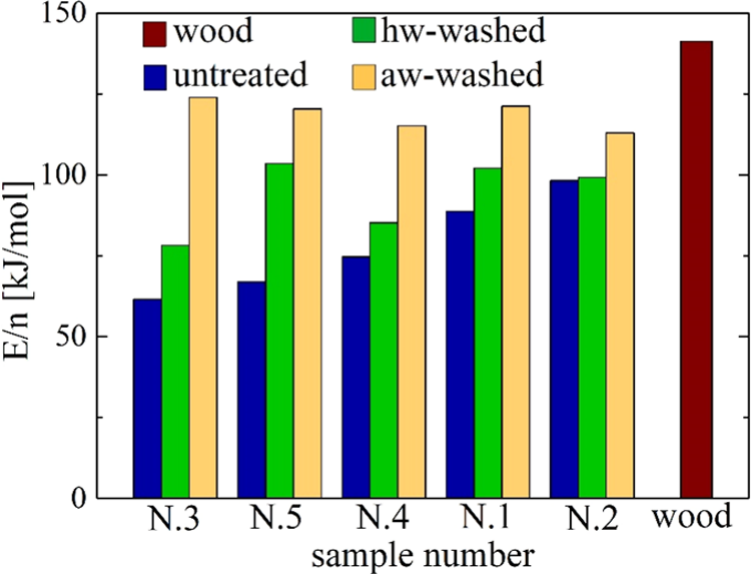
Histograms of the ratios of the activation energy to reaction order, *E*/*n*, of the global devolatilization reaction (data in Table 3) for the untreated, the hw-washed, and the aw-washed samples N.1 and 5 (the data is plotted from the least to the most aged sample). Beech wood data is included for comparison.

The results of the analytical evaluation of the ratio *E*/*n* for the global devolatilization reaction for the untreated and washed samples are reported in Table 3 and Figure 8. It is understandable that this evaluation essentially concerns the central zone of the thermogravimetric curves that in the absence of pretreatments represents the dynamics of cellulose–starch decomposition. In this case, the ratio *E*/*n* varies from 62 kJ/mol (sample N.3) to 98 kJ/mol (sample N.2); that is, it increases with sample aging. This finding is understandable as the cellulose content and crystallinity also increase, as testified by the successively higher peak rate and corresponding temperature.^57^ On the other hand, both features are described by higher activation energies.^61^ Values are much smaller than those obtained for beech wood (141 kJ/mol) and for microcrystalline or cotton linter celluloses^57^ (values of 209 and 198 kJ/mol, respectively). Agricultural residue variability gives rise to a variation in the *E*/*n* ratio of around 58%.

**Table 3:** Estimated Ratio Between the Activation Energy, *E*, and the Reaction Order, *n*, of the Global Devolatilization Reaction for the Untread, the hw-Washed, and the aw-Washed Samples N.1–5^a^

	*E*/*n* [kJ/mol]		
sample	untreated	hw	aw
N.3	61.6	78.2	123.9
N.5	67.0	103.5	120.3
N.4	74.7	85.2	115.1
N.1	88.7	102.1	121.2
N.2	98.2	99.2	112.9
beech wood	141.2		

a Beech wood data is included for comparison.

A significant increase in the *E*/*n* ratio is computed for the pretreated samples with value ranges of 78–104 kJ/mol (hw) and 113–124 kJ/mol (aw). The variability among the five samples is reduced to 32 and 10%. The aw treatment exerts a stronger effect on the *E*/*n* values, especially for the less aged samples (N.3 and N.5) with an increase between 15 and 101% (versus 1–54% for the hw treatment). Overall, following washing, the variability among the samples is reduced, and the contribution and crystallinity of cellulose are increased. Moreover, the behavior of the washed residues again approaches that of wood.

It is worth noticing that the kinetic parameters *E* and *n* are correlated; that is, an increase in *E* is also associated with an increase in *n.*(65) In other words, washing causes an increase in the *E* parameter significantly stronger than that represented by the ratio *E*/*n*. Information about the *n* values can be gained by looking at the characteristic temperature range Δ*T*_2_, considering the empirical correlation between the two parameters previously reported for char oxidation.^64^ It shows that *n* increases with Δ*T*_2_, and that for values of this below 50 K, it is comprised between about 0.5 and 1. Considering that, in the absence of treatment, Δ*T*_2_ roughly varies between 19 and 29 K, possible *n* values are around 0.6–0.7. After washing, Δ*T*_2_ is in the range of 23–39 K so that *n* is expected to vary in the range of 0.7–0.9.

### Thermograms and Global Pyrolysis Heats

**Figure 9:**
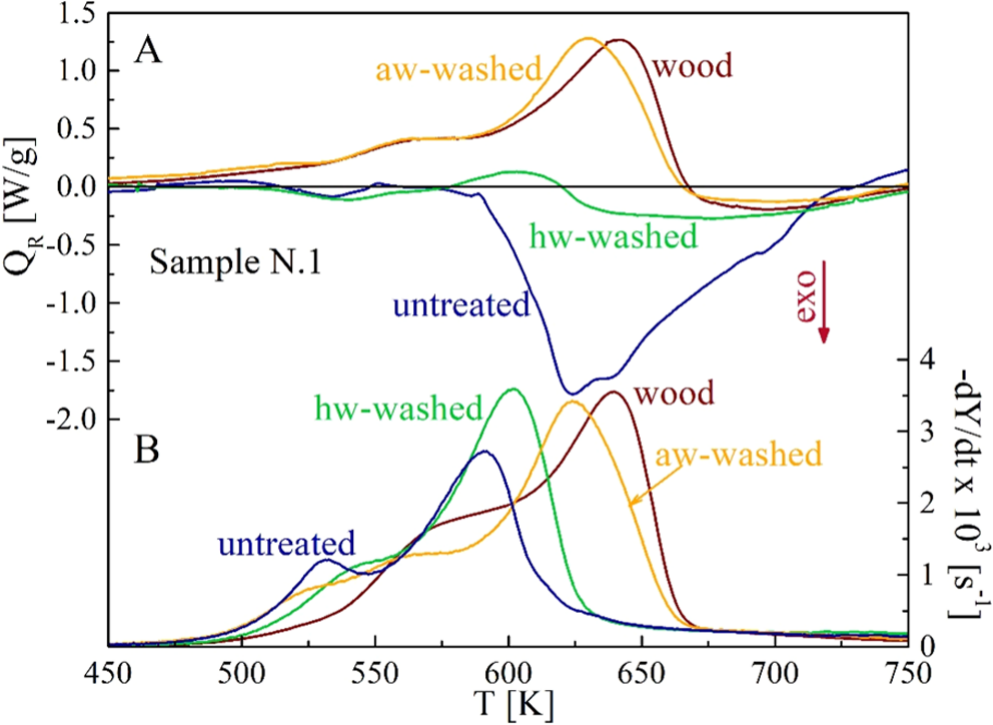
Calorimetric (A) and differential thermogravimetric (B) curves of the untreated, hw-washed, and aw-washed samples N.1 versus the heating temperature (heating rate 20 K/min). Beech wood curves are included for comparison.

Thermograms and weight loss curves are reported in Figure 9 for sample N.1 in the absence of treatment and for the hw and aw washing (beech wood data is also included). It is evident that both pretreatments act to reduce the global exothermicity of pyrolysis making the residues approach the wood behavior. In this regard, the acidic water treatment is more effective, as expected from the results of the thermogravimetric analysis. In the absence of pretreatment, the thermal decomposition of the sample N.1 is nearly thermally neutral for the first two reaction zones (decomposition of pectin–hemicellulose and cellulose–starch), while high rates of heat release are observed for the third zone (lignin–protein).

Following hw washing, the decomposition for the first zone remains almost neutral, but a moderate endothermicity appears for the second zone. The decomposition for the third zone is still exothermic, but the magnitude of the thermal event is highly reduced. When aw washing is carried out, the thermal behavior of sample N.1 becomes qualitatively and quantitatively similar to that of beech wood. Holocellulose decomposition (first two reaction zones) occurs endothermically, whereas a very moderate exothermicity appears only for the third zone (lignin). In fact, the globally endothermic decomposition of wood^49,50,66^ also shows moderate exothermicity only in correspondence to the tailing zone. Lignin (and proteins) are the main components responsible for char formation,^11,12,32^ which is an exothermic process. The extractives also produce high yields of charred solid residues,^25,58−60^ contributing to heat release. Moreover, primary and secondary charring reactions are also highly favored by alkali metals, thus further enhancing the exothermicity magnitude.^20,21^ Given the small sample mass and the use of open crucibles, it can be speculated that the observed thermal behavior mainly refers to primary reactions. These findings agree with previous DSC data,^50^ where extraction and water washing of an energy crop are reported to cause a progressive shift of the overall thermicity of the decomposition toward endothermicity.

**Figure 10:**
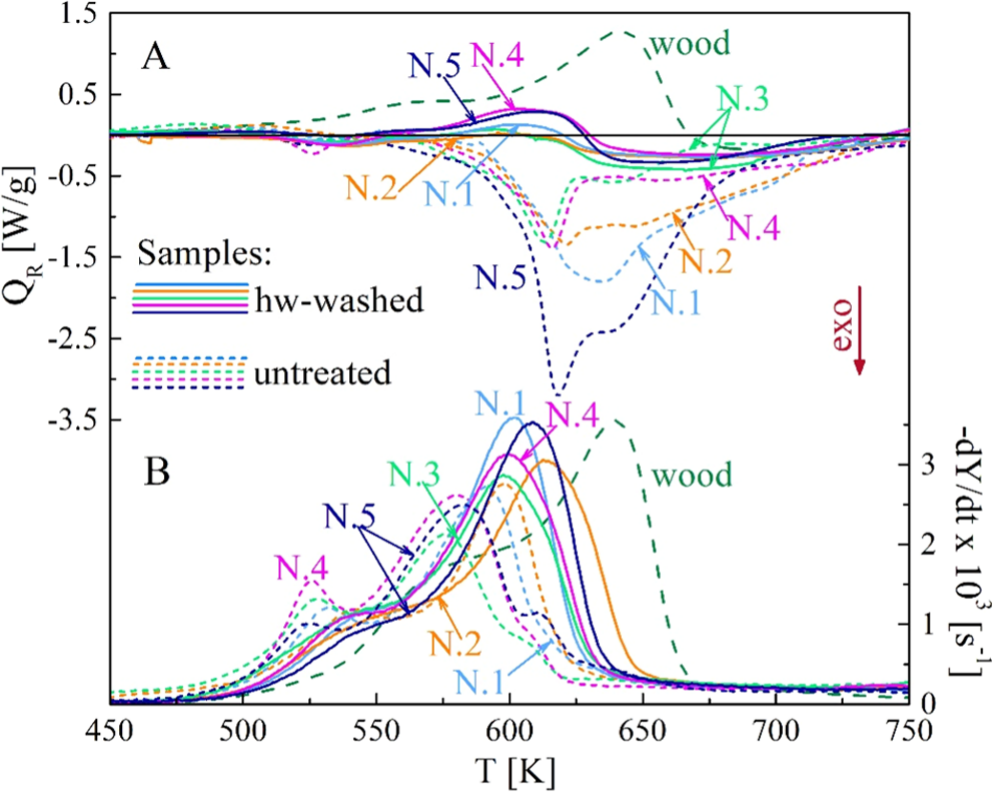
Calorimetric (A) and differential thermogravimetric (B) curves of the untreated and hw-washed samples N.1–5 versus the heating temperature (heating rate 20 K/min). Beech wood curves are included for comparison.

**Figure 11:**
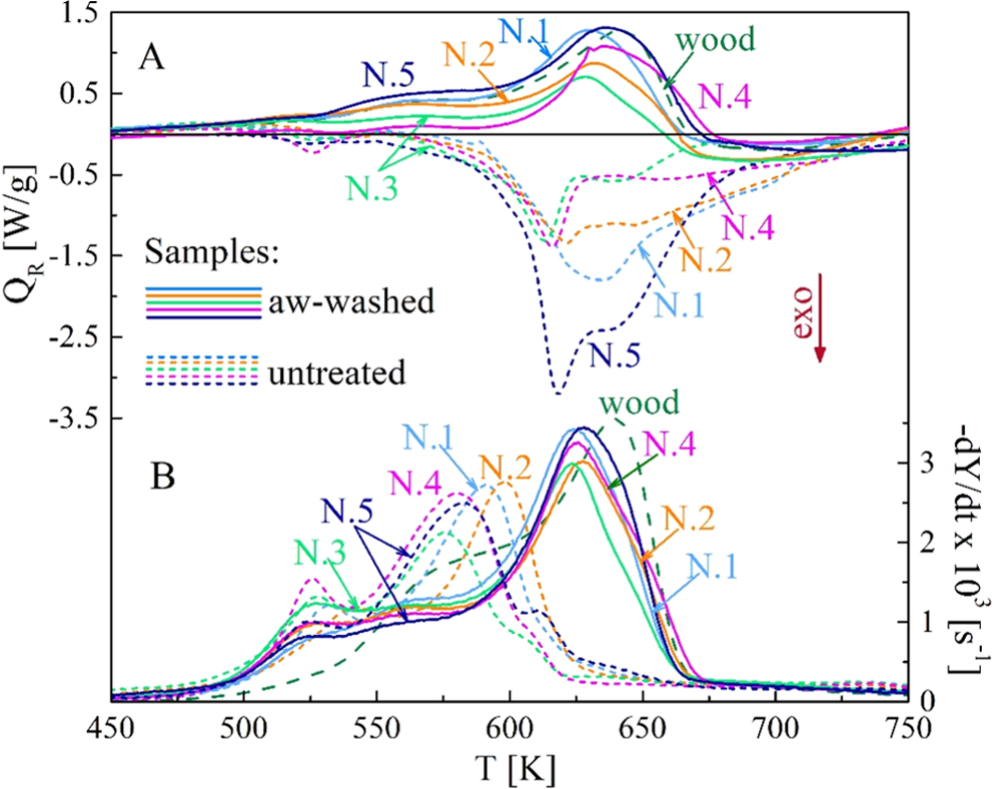
Calorimetric (A) and differential thermogravimetric (B) curves of the untreated and aw-washed samples N.1 and 5 versus the heating temperature (heating rate 20 K/min). Beech wood curves are included for comparison.

**Figure 12:**
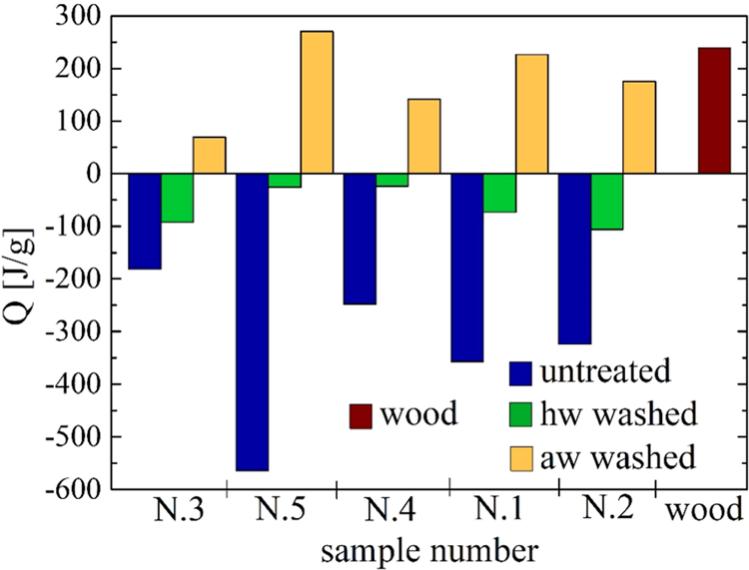
Histograms of the global reaction heat, *Q*, for the untreated, hw-washed, and aw-washed samples N.1–5 (the data is plotted from the least to the most aged sample). Beech wood data is included for comparison.

As shown in Figures 10–11, all the samples N.1–5 show the same qualitative trends, without and with washing, although the peak rates of the calorimetric curves are slightly different. A quantitative comparison can be made by considering the global reaction heat (Table 4 and Figure 12). The computation is made over the temperature range of 450–773 K. The (endothermic) heat for beech wood is 238 J/g, a value comparable with those of spruce^49^ and poplar^52^ woods. The exothermic behavior of the untreated samples is described by pyrolysis reaction heats varying from −181 J/g (sample N.3) to −564 J/g (sample N.5). The hot water treatment makes the conversion nearly thermally neutral with global pyrolysis heats varying from about −106 J/g (sample N.2) to −24 J/g (sample N.4). Acidic water washing shifts from the exothermic to endothermic process with global reaction heats from 69 J/g (sample N.3) to 270 J/g (sample N.5).

**Table 4:** Computed Global Reaction Heats for the Untreated, hw-Washed, and aw-Washed Samples N.1–5^a^

	*Q* [J/g]		
sample	untreated	hw	aw
N.3	–181	–91	+69
N.5	–564	–26	+270
N.4	–248	–24	+141
N.1	–357	–73	+226
N.2	–324	–106	+175
beech wood	+238		

a Beech wood data is included for comparison.

These results clearly indicate that the washing pretreatment is apt to modify not only the decomposition characteristics but also the energetic aspects of the process. The removal of both alkali metals and nonstructural components (extractives, pectin, starch, and proteins) reduces the char yields and thus reduces or eliminates the conversion exothermicity. Therefore, similarly to the results already discussed for the decomposition rates, the thermal behavior of the washed samples closely approaches that of wood, especially for the acidic treatment.

## Conclusions

Microscale analysis (thermogravimetry and calorimetric analysis) is carried out to ascertain the role of hot water and acidic washing on the widely different properties of five samples of potato stem waste, using beech wood for comparison. It is found that washing always acts to reduce the variability of the wastes, making their behavior approach that of wood. This finding is due to the removal of nonstructural inorganics (AAEMs) and organics (pectin, starch, and protein) present in large amounts in the untreated residues.

Following the pretreatments, crop residue decomposition tends to occur at higher temperatures and with higher rates and reduced char yields, especially for acidic water pretreatment. The exothermic character of the decomposition reaction also becomes weaker (hot water washing) and then turns into an endothermic character (acidic water). In this way, thermogravimetric curves (devolatilization rates) and calorimetric curves (global reaction heat) become almost coincident with those obtained for beech wood. In conclusion, from a quantitative point of view, acidic water washing is an effective method for making the waste properties more uniform and comparable with those of woody materials.

In addition to quantitative information about the effects induced by washing on the devolatilization characteristics and global reaction heats, an analytical evaluation of the ratios between the activation energy and reaction order has been made. It is shown that they increase, again approaching the wood value, especially for the acidic treatment (*E*/*n* around 113–124 kJ/mol versus 141 kJ/mol for wood). Hence, the cellulose contents, and most likely the crystallinity index, increase with washing treatment.

The better effectiveness of acidic water washing, for reducing the variability of wastes and making their decomposition behavior approach that of wood, avoids the solvent preheating required by hot water. There is, however, the disadvantage of generating an acidic leachate byproduct, which should be disposed of or treated for possible reuse. This is an aspect that requires further investigation in relation to the use of alternative organic acidic substances.

## Supporting Information Available

Definitions of the TGA parameters (PDF)
