# Supplementary material for: Demineralization Pretreatments for Reducing Biomass Variability in Pyrolysis
Source: ACS Omega. 2024 Feb 13;9(8):9536–46. doi: 10.1021/acsomega.3c09321 (PMC10905592; doi:10.1021/acsomega.3c09321)
Supplement: Supplementary file 1 — ao3c09321_si_001.pdf [file ao3c09321_si_001.pdf]

DEMINERALIZATION PRETREATMENTS FOR REDUCING BIOMASS VARIABILTY IN PYROLYSIS

Carmen Branca,[1*] Colomba Di Blasi[2]

[1] Istituto di Scienze e Tecnologie per l'Energia e la Mobilità Sostenibili (STEMS), C.N.R., P.le V. Tecchio, 80125 Napoli, Italy
[2] Dipartimento di Ingegneria Chimica, dei Materiali e della Produzione Industriale, Università degli Studi di Napoli "Federico II", P.le V. Tecchio, 80125 Napoli, Italy

* Corresponding author: e-mail: carmen.branca@stems.cnr.it; tel:39-081-7682232

Graphical Definition of the Thermogravimetric Parameters

The thermogravimetric parameters [25,28,46,64] are represented in Fig. SM1 (untreated sample N.1) and Fig. SM2 (aw washed sample N.2): the peak rate, $-dY_{peak}/dt$, and the peak/shoulder rate of the first or third zone, $-dY_{ps1}/dt$ or dY_{ps2}/dt, with the corresponding temperatures T_{peak} and T_{ps1} or T_{ps2} and mass fractions, Y_{peak}, and Y_{ps1} or Y_{ps2}, the temperature range fwhm (the full width of the rate curve at the half maximum), the final charred residue (mass fraction) at a temperature of 773K, Y_{773}, and the characteristic temperature ranges, ΔT_1 and ΔT_2.

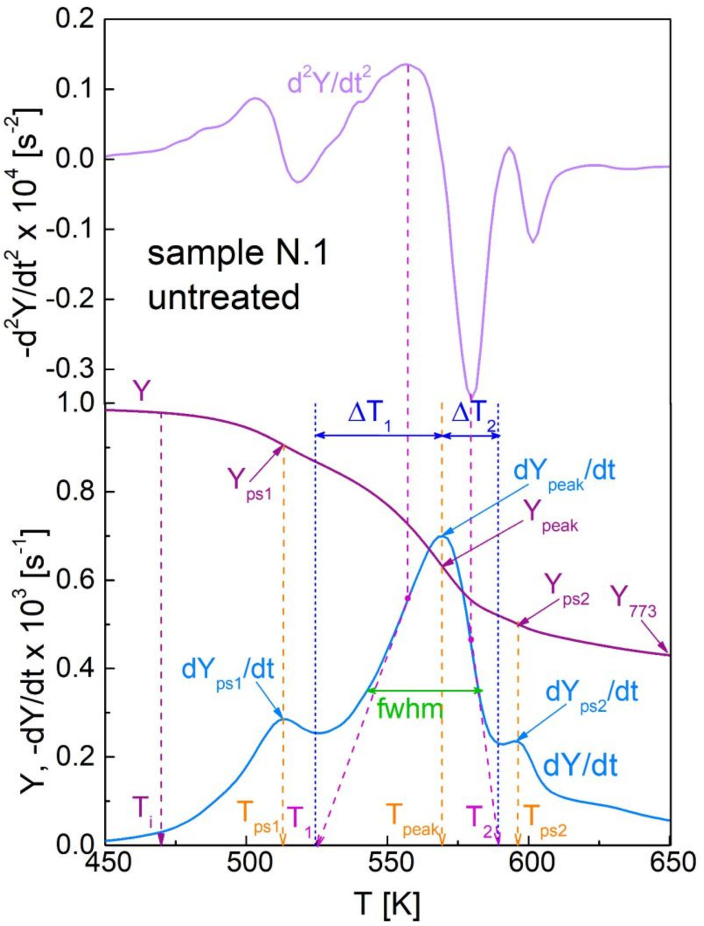

Fig. SM1 – Definition of thermogravimetric parameters: example for untreated samples (sample N.1).

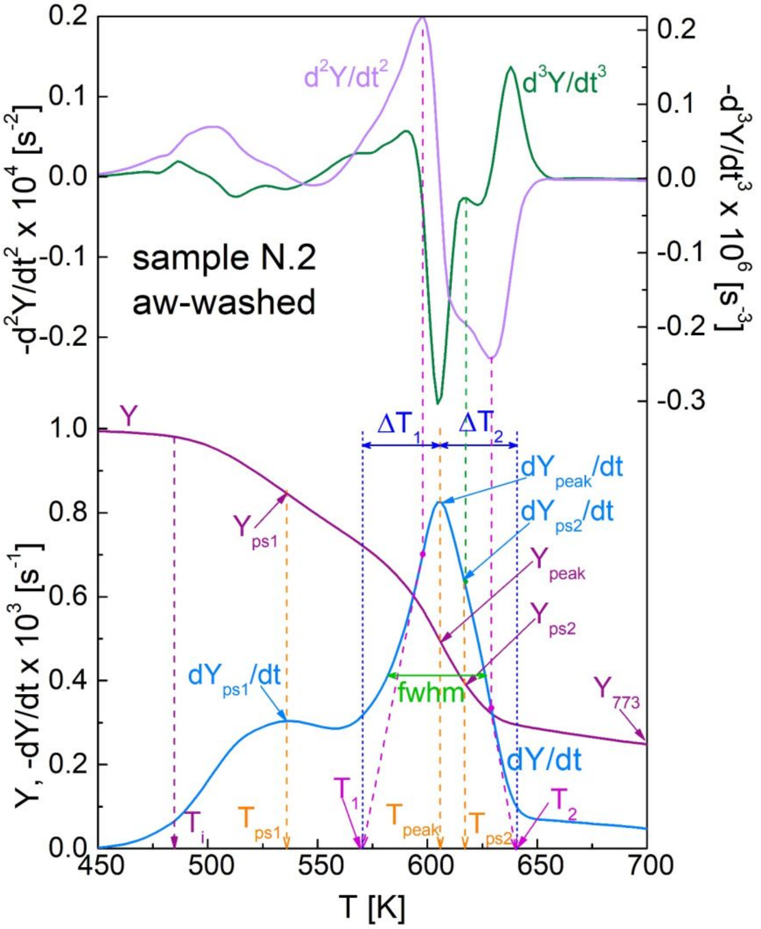

Fig. SM2 – Definition of the thermogravimetric parameters: example for aw washed samples (sample N.2).
